# Pancreatic carcinoma-specific immunotherapy using novel tumor specific cytotoxic T cells

**DOI:** 10.18632/oncotarget.13469

**Published:** 2016-11-19

**Authors:** Jianjun Lei, Zheng Wu, Zhengdong Jiang, Jiahui Li, Liang Zong, Xin Chen, Wanxing Duan, Qinhong Xu, Lun Zhang, Liang Han, Qingyong Ma, Zheng Wang, Dong Zhang

**Affiliations:** ^1^ Department of Hepatobiliary and Pancreas Surgery, First Affiliated Hospital of Medical College, Xi'an Jiaotong University, Xi'an 710061, Shaanxi Province, China

**Keywords:** tumor specific cytotoxic T lymphocytes, immunotherapy, pancreatic carcinoma

## Abstract

Pancreatic cancer represents one of the most lethal human cancers. Investigation of the effective targeting to the tumor cells is essential for both primary tumors and metastases. Tumor specific cytotoxic T lymphocytes (CTLs) have recently been considered to be the attractive vehicles for delivering therapeutic agents toward various tumor diseases. This study was to explore the distribution pattern of CTL carrying the lentiviral vectors with the characteristic of adenoviral E1 gene under the control of the cell activation-dependent CD40 ligand promoter (Lenti/hCD40L/E1AB). Following transduction with adenoviral particles containing chimeric type 5 and type 35 fiber proteins (Ad5/35-TRAIL), these CTLs produced infectious virus when exposed to SW1990 cells. We found that the novel CTL harboring Lenti/hCD40L/E1AB and Ad5/35-TRAIL inhibited pancreatic cancer cell growth and angiogenesis *in vitro* and *in vivo*. Furthermore, Ad5/35-TRAIL transduced CTL induced significant apoptosis in pancreatic carcinoma cell lines and upregulated IFN-gamma (IFN-γ) secretion of CTLs. Importantly, Ad5/35-TRAIL transduced CTLs had no inhibitory effect on normal cells. Thus, the novel CTLs may be safe and feasible for the development of gene therapy approaches to pancreatic carcinoma.

## INTRODUCTION

Accompany with a 5-year overall survival rate less than 7%, pancreatic cancer is the fourth leading cause of cancer related deaths in the United States [1]. The only curative treatment for pancreatic cancer is pancreaticoduodenectomy. Only 10 to 15% will be resectable, and the remainder will be locally advanced unresectable disease as well as an incidence of unrecognized metastases. Gemcitabine is the most effective chemotherapy drug for advanced pancreatic cancer, improving survival rate as compared with 5-fluorouracil (5-FU) [2, 3]. However, recent trials showed that gemcitabine failed to increase survival when combined with other chemotherapeutic drugs [2, 4]. Therefore, it is urgent needed to find a new therapy for advanced pancreatic cancer. Cancer cells including pancreatic cancer cells could be recongnized by CTLs [5, 6]. Tumor infiltrated with CTLs and T helper cells is a favourable prognostic factor for pancreatic cancer [7]. Tumor-reactive T cells, isolated from blood of cancer patients, are capable of tumor rejection [8].

CTLs are highly effective in infiltrating tumor sites at multi organs as shown in a number of preclinical and clinical models [9, 10]. However, while CTLs may eliminate some kinds of tumors, many malignancies succeed escaping from the effective attack due to some evasion mechanism [11, 12]. Therefore, immunotherapy combined with virus therapy is an effective treatment for pancreatic cancer. The interaction between CD40 and CD40 ligand (CD40L) could trigger DC activation. CD40L is only transiently expressed on the surface of activated CD4+ T cells for less than 24 hours [13]. CD40 expression could induce the maturation of CD40+ dendritic cell and B lymphocytes. The CD40L promoter is closely mediated by the AT hook transcription factor AKNA. AKNA only expressed transiently following antigen-activated T cell [14, 15]. So, if this promoter were used to activate the adenoviral E1 gene, expression would be triggered only following the T cell encountered its target.

If the Ad5 (coxsackievirus–adenovirus) vectors carry fibers from species B Ad serotype 35 (Ad5/35), they could infect cells through CD46, which usually expressed in most undifferentiated cells [16, 17]. In this study, we used the Ad5/35 vector, which is CAR independent and could infect human T cells [18]. Tumor Necrosis Factor Related Apoptosis Inducing Ligand (TRAIL) exhibits prominent pro-apoptotic effect among multiple malignant cell types. It is supposed to be a highly promising anticancer agent. Especially, TRAIL essentially has no effect on normal cells [19]. Through extrinsic apoptotic mechanism, TRAIL triggers its receptors and accumulates caspase 8, which is then evolved into its active form. The active form of caspase 8 then cleaves Bid (the BH3-only molecule), which then have a reciprocal action with mitochondrial anti- and pro-apoptotic elements.

Here, we constructed a new CTL carrying LVCD40Lpr and Ad5/35-TRAIL which resulted in angiogenesis and growth inhibition and obvious apoptosis in pancreatic cancer cell lines. This kind of CTL maybe used as a promising treatment for advanced pancreatic cancer.

## RESULTS

### CD40L/E1 transduction does not influence CTL proliferation

**Figure 1:**
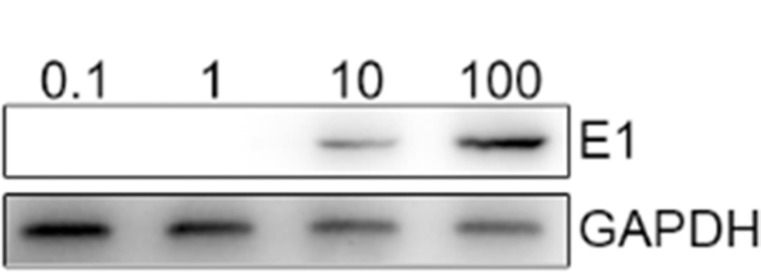
E1a protein is detected in CTL after infected with different MOI of adenoviruses Western blot analysis: The CTLs were infected with 0.1, 1, 10 and 100 MOIs of adenoviruses, then lysed, and the proteins were separated on a gel. E1a monoclonal antibody was used to detect the target protein.

We first used a CD40L/E1 lentivirus that carrying the adenovirus E1 gene to transduce the CTL (CD40L/E1-CTL), the schema was described in methods, and western blot was used to detect the suitable MOIs of CD40L/E1-CTL. CTLs were infected with the different CD40L/E1 MOIs for 48 h, and then the E1 adenovirus protein expression was analyzed by western blot (Figure 1). The CTLs were infected with different MOIs of adenoviruses (0.1, 1, 10 and 100). According to the results, we determined that 50 MOIs was the suitable MOIs.

**Figure 2:**
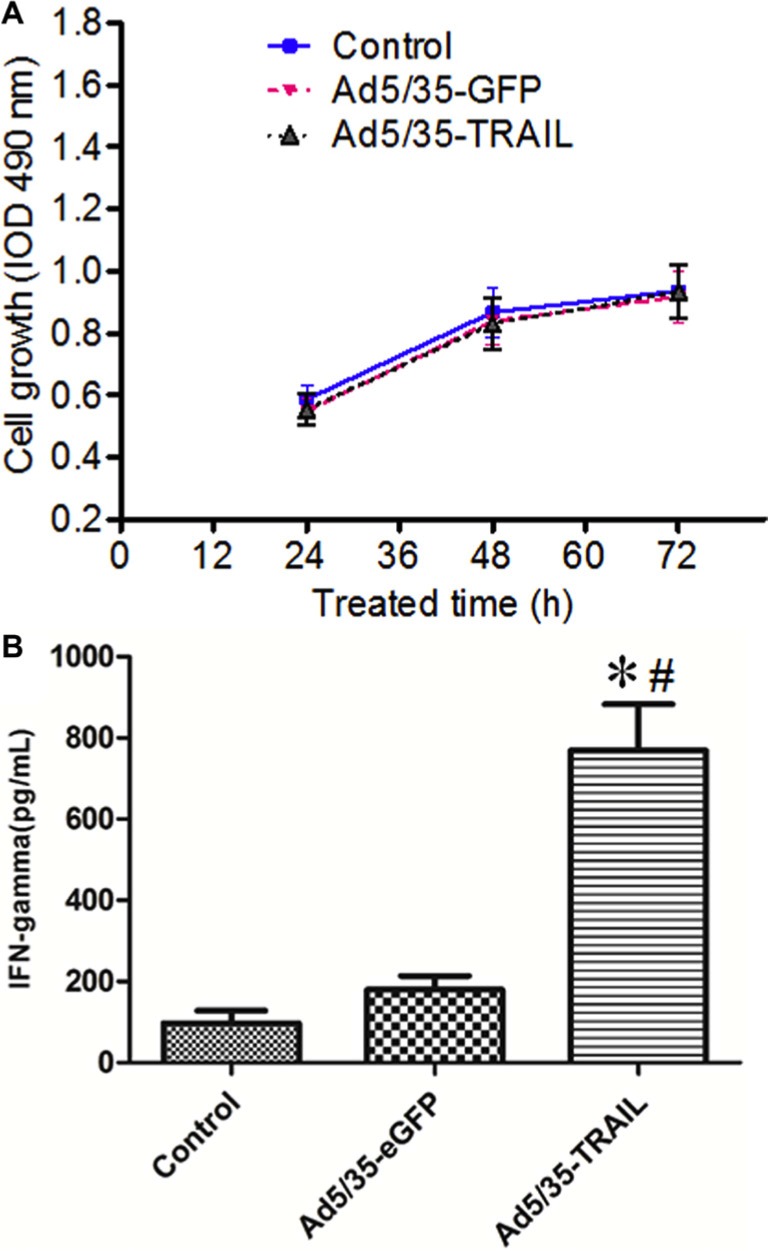
Adenovirus-transduced CTL function (**A**) The graphs illustrate the proliferation rate of non-transduced and transduced CTLs (CD40L-CTLs infected with Ad5/35-GFP or Ad5/35-TRAIL) cultured in minimum media (Yssel pluse 10% FCS) alone by MTT method. (**B**) IFN-γ level in the supernatant of non-transduced and transduced CTLs were detected by ELISA. *P < 0.05 compared with non-transduced group; ^#^*P* < 0.05 compared with Ad5/35-GFP -transduced group.

To measure the proliferative ability of adenovirus-transduced CD40L/E1-CTL, the MTT assay was used to analyze. CD40L/E1-CTLs were transduced with Ad5/35-GFP or Ad5/35-TRAIL for 24, 48 and 72 h, there were no statistical significance among three groups (Figure 2A) (*P* > 0.05). The CD40L/E1-CTLs transduced with Ad5/35-TRAIL (Ad-TRAIL-CD40L/E1-CTL) exhibit similar proliferative capability as compared with control and Ad-eGFP-CD40L/E1-CTL groups.

### Ad5/35-TRAIL transduction upregulates IFN-γ secretion in CD40L/E1-CTL cell

IFN-γ levels in Ad-CD40L/E1-CTL cell culture supernatants were determined by ELISA (Figure 2B). The supernatants collected from CD40L/E1-CTL, Ad-eGFP-CD40L/E1-CTL and Ad-TRAIL-CD40L/E1-CTL cells cultured for 48 h, Ad-TRAIL-CD40L/E1-CTL group had significantly higher amounts of secreted INF-gamma compared to levels in supernatants obtained from other groups (*P* < 0.05).

### Ad-TRAIL-CD40L/E1-CTL inhibits angiogenesis *in vitro*

**Figure 3:**
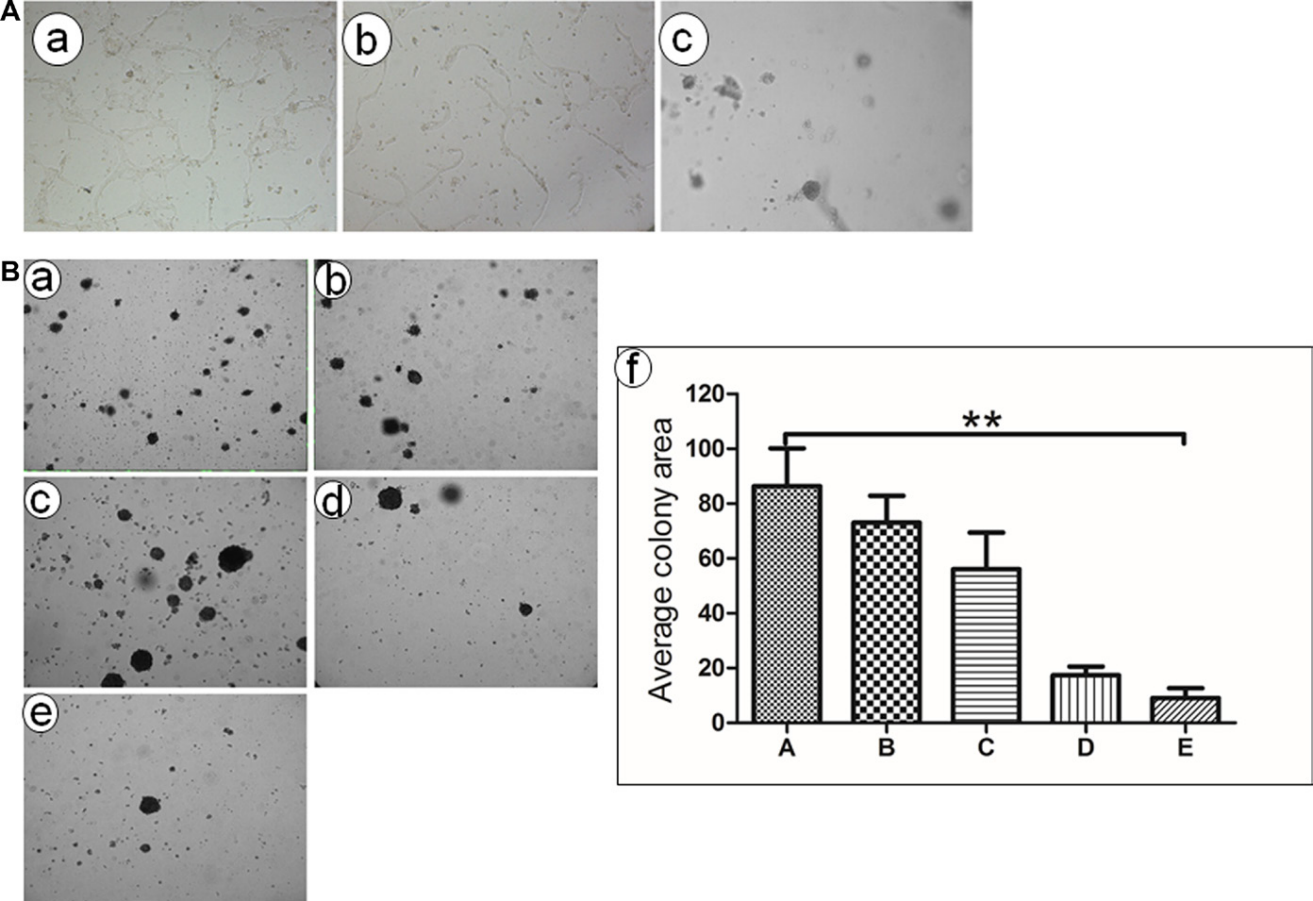
Tube formation and colony formation detection *in vitro.* (**A**) *In vitro* angiogenesis assay showed that HUVEC formed vessel like structures (tubes) when plated on ECM wells. (a) Untreated HUVEC cells; (b) HUVEC cells treated with CD40L-CTLs infected with Ad5/35-GFP adenovirus; (c) HUVEC cells treated with CD40L-CTLs infected with Ad5/35-TRAIL; (**B**) Ad5/35-TRAIL-CD40L/E1-CTL decreases colony formation of pancreatic carcinoma cell line SW1990 in soft agar. (a) Untreated SW1990 cells grown in the soft aga; (b) SW1990 cells treated with CTLs; (c) SW1990 cells treated with CTLs treated with Ad-CD40L;(d).SW1990 cells treated with Ad5/35-GFP-CD40L adenovirus. (e) SW1990 cells treated with Ad5/35-TRAIL-CD40L adenovirus. **P < 0.01 compared with Untreated group.

To determine the effect of Ad-TRAIL-CD40L/E1-CTL on angiogenesis *in vitro*, we selected the HUVEC co-culture system to observe the vessel like structures or network formation (Figure 3A). The results showed that Ad-TRAIL-CD40L/E1-CTL apparently inhibited the HUVEC vessel like structures formation, compared with control groups. Additionally, the Ad-TRAIL-CD40L/E1-CTL also significantly suppressed tube formation as compared to Ad-eGFP-CD40L/E1-CTL group.

### Ad-TRAIL-CD40L/E1-CTL suppresses pancreatic cancer cell growth

We also used colony formation assay to measure the effect of Ad-TRAIL-CD40L/E1-CTL on SW1990 cell growth in soft agar (Figure 3B). As shown in results, the colony-forming ability of SW1990 cells was prominently inhibited in Ad-TRAIL-CD40L/E1-CTL group as compared with CTL and CD40L/E1-CTL group. The number of colonies strongly decreased in soft agar. So Ad-TRAIL-CD40L/E1-CTL could apparently decrease the growth and oncogenic behavior of SW1990 cells.

### Ad-TRAIL-CD40L/E1-CTL has no inhibitory effect on human normal cells

**Figure 4:**
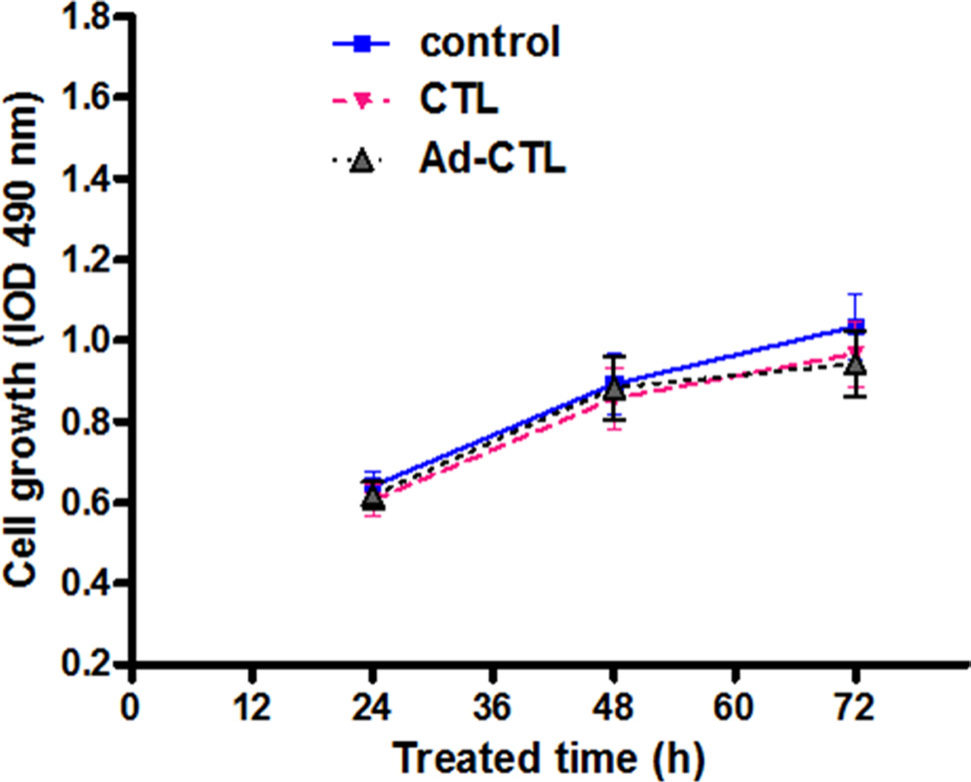
The effect of Ad5/35-TRAIL-CD40L-CTL (Ad-CTL) on normal HFF cell proliferation Ad-CTL has no inhibitory effect on human normal cells.

To investigate whether Ad-TRAIL-CD40L/E1-CTL have an effect of killing human normal cells, we tested the HFF cells (human foreskin fibroblast cell line) proliferative activity co-cultured with CTL and Ad-TRAIL-CD40L/E1-CTL cells by MTT assay. CTL and Ad-TRAIL-CD40L/E1-CTL cells were co-cultured with HFF cells respectively for 24, 48 and 72 h, the results showed that there were no difference in the proliferation rate among the same for three groups (Figure 4). The different groups had no statistical significance (*P* > 0.05), and the Ad-TRAIL-CD40L/E1-CTL cells has the no inhibitory effect on HFF cells compared with controls.

### Ad-TRAIL-CD40L/E1-CTL inhibits the tumor growth *in vivo*

**Figure 5:**
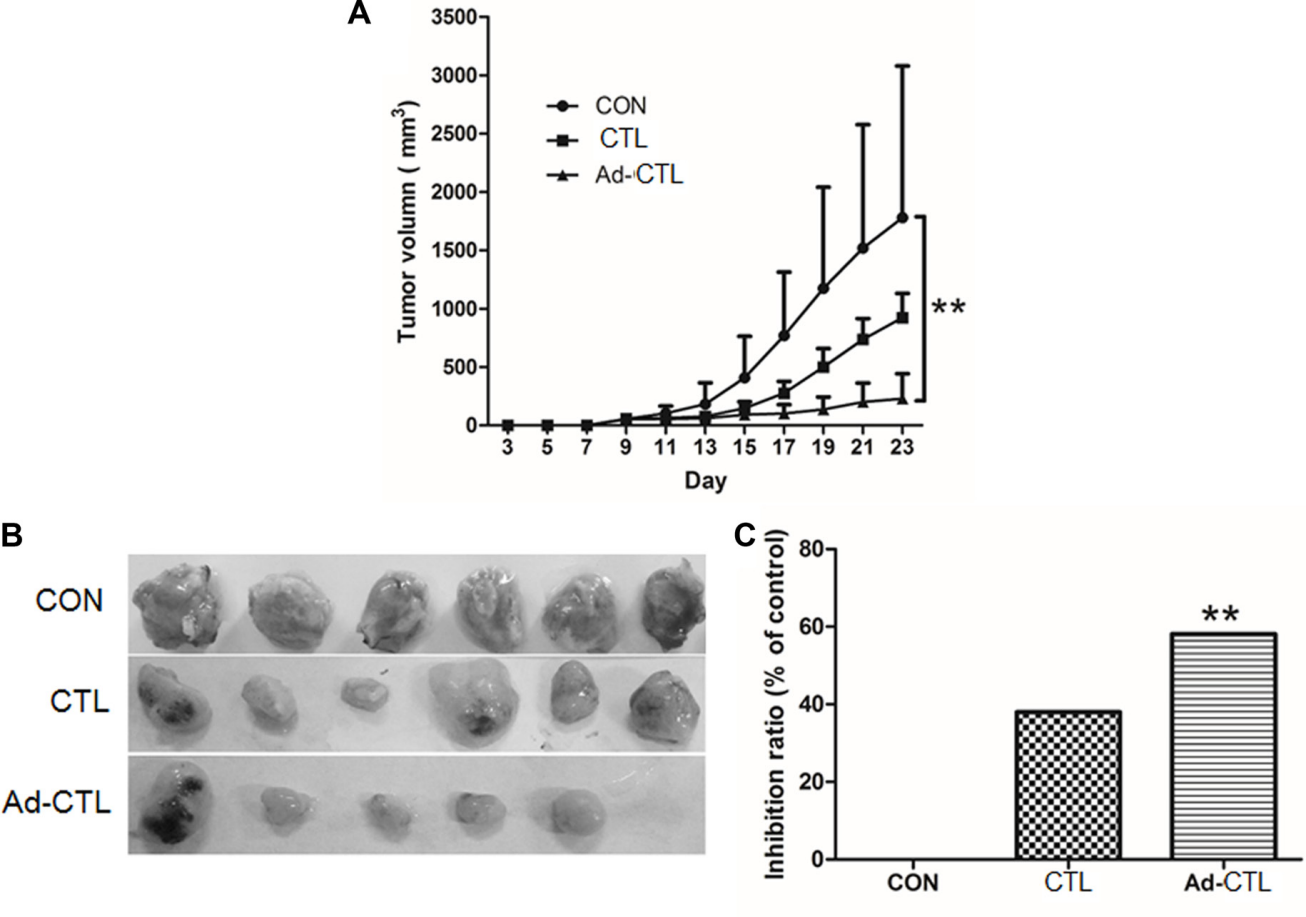
Ad5/35-TRAIL-CD40L-CTL (Ad-CTL) inhibits pancreatic carcinoma growth *in vivo* Control group (SW1990 cells), CTL group (SW1990 + CTL), and Ad-CTL group (SW1190 + Ad-CTL) were injected s.c. into the right flank of nude mice. (**A**) Tumor growth curve of different group. (**B**) Representative photograph of tumor sizes in different groups. (**C**) Statistical significance was determined using the ANOVA test versus control group. **P < 0.01.

To further elucidate whether Ad-TRAIL-CD40L/E1-CTL also inhibits tumor growth *in vivo*, the CTL cells were subcutaneously injected into the tumor xenografts derived from SW1990 cells in BALB/c nude mice. There was an obvious difference in the growth of tumors injected with Ad-TRAIL-CD40L/E1-CTL cells compared with tumors injected with CTL cells or 0.9% NaCl (Figure 5). Ad-TRAIL-CD40L/E1-CTL significantly inhibited tumor growth as compared with control group.

### Ad-TRAIL-CD40L/E1-CTL restrains pancreatic cancer angiogenesis and promotes cancer cell apoptosis *in vivo*

**Figure 6:**
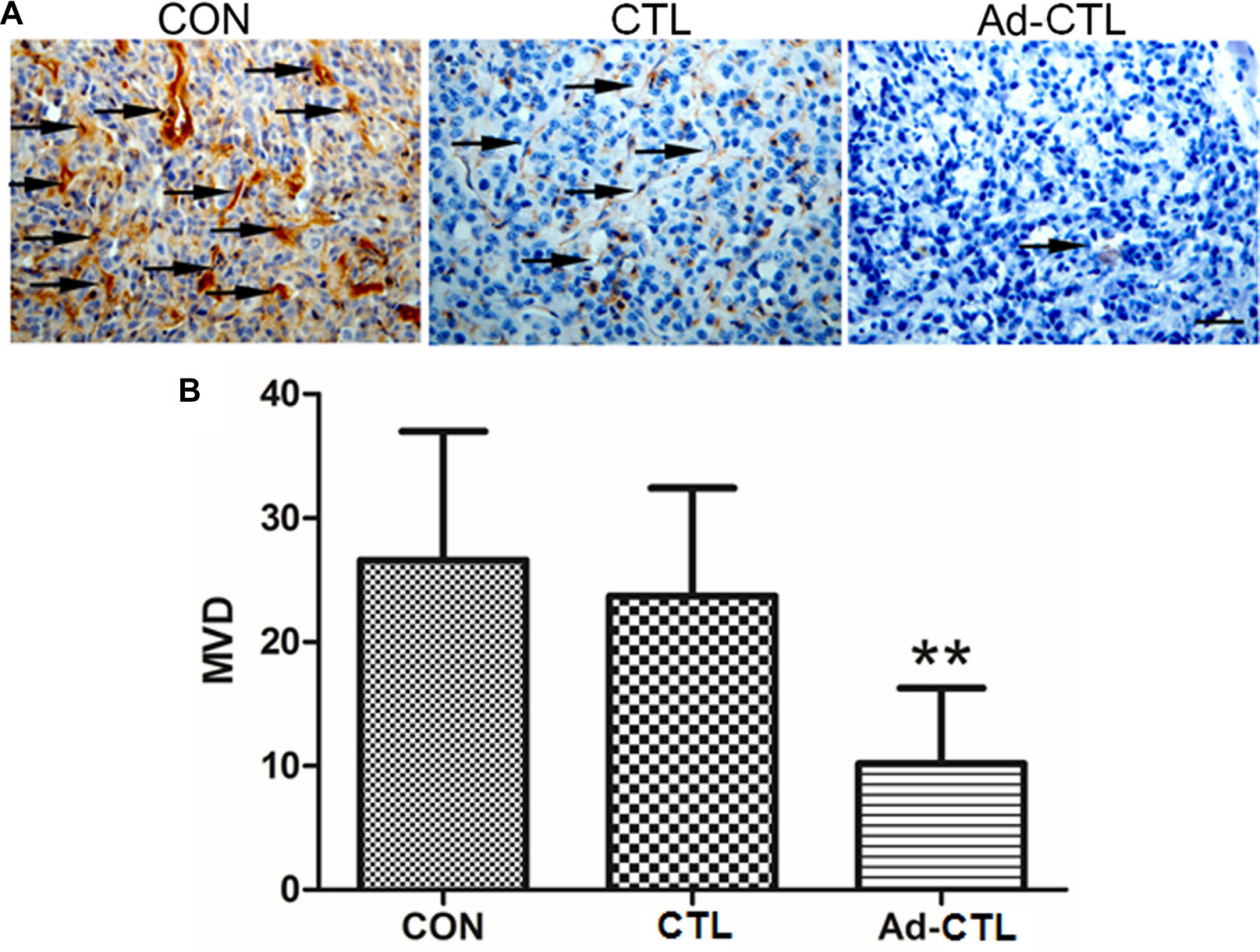
The effect of Ad5/35-TRAIL-CD40L-CTL (Ad-CTL) on pancreatic carcinoma angiogenesis *in vivo* (**A**) Representative photograph of CD31 staining in different groups. (**B**) Statistical analysis of microvessel density in different group. **P < 0.01 compared with control group.

To investigate whether Ad-TRAIL-CD40L/E1-CTL could inhibit angiogenesis *in vivo*, we detected CD31 expression in stained paraffin sections from tumor xenografts. CD31 is a specific endothelial cell marker which has been commonly used for microvessel quantification in tumors. Microvessel density (MVD) of tissue section is considered an index of neovascularization. The MVD of tumor derived from Ad-TRAIL-CD40L/E1-CTL group was significantly lower than that of control group (Figure 6). These findings indicate that the inhibition of tumor xenograft growth by injected Ad-TRAIL-CD40L/E1-CTL might be due to suppression of angiogenesis.

**Figure 7:**
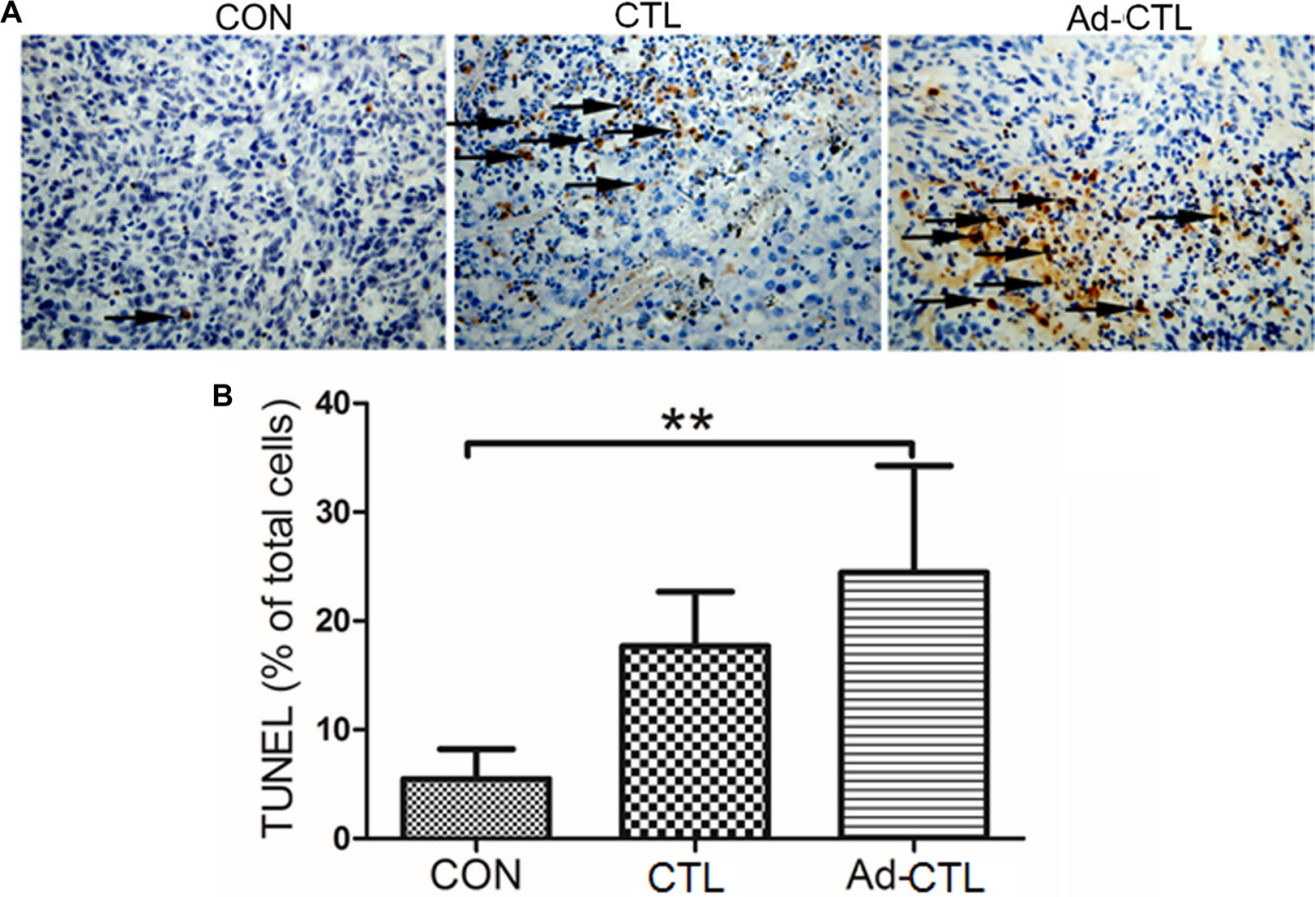
The effect of Ad5/35-TRAIL-CD40L-CTL (Ad-CTL) on pancreatic carcinoma cell apoptosis *in vivo* (**A**) Representative photograph of TUNEL assays in different groups. (**B**) Statistical analysis of apoptosis in different group. **P < 0.01 compared with control group.

Induction of apoptosis by Ad-TRAIL-CD40L/E1-CTL in tumor derived from SW1990 cells was detected by TUNEL assay (Figure 7). There was a prominent increase in the number of TUNEL-positive pancreatic cancer cells in Ad-TRAIL-CD40L/E1-CTL group as compared with the untreated control group, which indicate that Ad-TRAIL-CD40L/E1-CTL cells treatment could significantly increase tumor cell apoptosis as compared with untreated control cells.

## DISCUSSION

Pancreatic cancer represents the fourth commonest cause of cancer-related mortality in the United States. However, no adequate therapy for pancreatic cancer has yet been found. Standard therapy includes surgery, chemotherapy (mostly with gemcitabine), and radiotherapy, but much effort is made to develop new molecular therapies [20, 21]. Qiu et al. showed that synthesized α1,3-galactosyl epitope-pulsed dendritic cells (DCs) along with CTL cells may be used as a new immunotherapy for pancreatic cancer [22]. In this pilot study, fourteen patients with advanced pancreatic cancer were enrolled and treated with gemcitabine combined with oxaliplatin and radiotherapy. DCs and CTLs were injected into the patients. The data showed that the procedure was safe without serious side effects. 12 patients developed strong positive delayed-type IV hypersensitivity to the autologous cancer cell lysate and had robust systemic cytotoxicity elicited by IFN-γ expression secreted by peripheral blood mononuclear cells. Another study showed that adoptive immunotherapy using *ex vivo* expanded, cytokine-induced killer (CIK) cells in gemcitabine-refractory advanced pancreatic cancer had comparable progression-free survival (PFS) and overall survival (OS) to survival data of previous trials that assessed conventional chemotherapies while maintaining tolerability [23].

In this study, our data showed that Ad-TRAIL-CD40L/E1-CTL inhibited pancreatic cancer cell growth and angiogenesis *in vitro* and *in vivo*, and induced higher level of IFN-γ secretion in CTL, however had no side effects on normal cells, which suggest that using targeted adenoviral particles delivered by cytotoxic T cells is a promising therapeutic approach for cancer.

Previous studies have shown that CTLs could directly kill some malignant cells [24], which express specific antigenic peptides, often referred to as CTL epitopes, in the context of specific class I MHC molecules [25, 26]. These tumor-associated antigenic peptide could be used as a peptide-based vaccine to amplify the anti-tumor CTL response [27]. However, when these peptide is from non-mutated differentiation antigens, it is not sufficient to simulate persistent and robust anti-tumor CTL responses [28, 29]. This is due to immune tolerance mechanisms which generally inhibit or remove high activity auto-reactive T cells [30]. Therefore, bulk tumor-specific CTL, especially those which recognize non-mutated tumor-specific antigens, are removed at the thymus and at the periphery. As a result, little tumor-specific CTL remains [31, 32].

In this study, when the CTLs (transduced with both E1 lentivirus and E1-deficient Ad5/35 adenoviral particles) encounter with target cells, they could generate abundant infectious adenoviral vector. Though, according to a CTL-restricted receptor pathway, the transduction of target tumor cells was substantially lower when CTLs encounter CTL targets, consistent with a lower degree of T-cell activation. These abundant adenoviral vector was sufficient to infect a high proportion of CTL positive target cells. If these vectors carry a potential anti-tumor gene (such as TRAIL), then it was capable to eliminate the target cells. Therefore, Ad-CD40L-CTL could cause much greater tumor cell death than the use of cytotoxic T cells alone [33].

In summary, CTLs (transduced with both E1 lentivirus and E1-deficient Ad5/35 adenoviral particles), incorporation of a vector encoding a potentially oncolytic gene, such as TRAIL, is a promising therapeutic approach for pancreatic cancer treatment.

## MATERIALS AND METHODS

### Cell culture

The human pancreatic carcinoma cell line SW1990, human embryonic kidney cell line HEK293T and human foreskin fibroblast cell line HFF were obtained from the American Type Culture Collection (Rockville, MD), and cultured in Dulbecco's modified Eagles medium (DMEM) supplemented with 10% FBS and 100 IU/ml penicillin/streptomycin in a 37°C humidified incubator with 5% CO_2_.

### Production of lentiviral vectors

The CD40L promoter was cloned from human genome and subcloned into the lentiviral shuttle vector pLenti, named pLenti/hCD40L. The specific primers of CD40L promoter: upstream 5′- CCCAAGCTTAAGAAAGCAGGTGCTAACTATATAG-3′ and downstream 5′- CGGGATCCGCTGTGTTAAAGTT GAAATGGTATC-3′. The E1 gene was amplified by polymerase chain reaction and subcloned into the downstream of pLenti/hCD40L vector, pLenti/hCD40L/E1. Lentiviral particles were produced by performing transient co-transfection in HEK293T cells and concentrated through ultracentrifugation [34].

### Production of adenoviral particles

According to the our previously reports [34], to constructed the Ad5/35-GFP and Ad5/35-TRAIL vectors, the green fluorescent protein (GFP) and IRES-TRAIL gene were inserted into pAd35CMV downstream of the cytomegalovirus (CMV) promoter. And then we co-transfected the plasmid together with pAd35Helper into HEK293T cells to generate recombinant virus (Ad5/35-TRAIL). Adenoviral particles were purified by ultracentrifugation in CsCl gradients.

### Lymphocytes isolation and identification

Lymphocytes isolation and identification was described as our previous study [34]. Briefly, Lymphocytes was isolated from human blood using the human lymphocyte separation medium, then transferred to a 25 cm^2^ plate (Costar), precoated with RetroNectin (2 mg/L; TaKaRa, Japan) and anti-CD3 antibody (50 ng/ml; TaKaRa, Japan) at 1 × 10^6^ cells per well, and incubated for 48 hours for optimal activation before transduction. The stimulated CTL lines were resuspended at 1 × 10^6^ cells/mL in complete medium supplemented with IL-2 (300 U/ml), IFN-c (400 U/ml), phytohaemagg lutinin (10 U/ml), rhIL-4 (100 U/ml), GM-CSF (10 U/ml) and rhIL-2 (100 IU/mL), and then incubated for 36 hours at 37°C and 5% CO_2_. CD3-FITC, CD4-PE, CD8-PE, CD56-PE, CD226-FITC, CD11-PE and CD305-FITC monoclonal antibodies (MAbs) with the appropriate fluorescein isothiocyanate (FITC)- or R-phycoerythrin (PE)-conjugated were purchased from BD Bioscience (Mountain View, CA). Cells were washed and stained with the appropriate antibodies for 20 minutes at 4°C in the dark in phosphate-buffered saline (PBS) supplemented with 0.1% bovine serum albumin (BSA). Control cells were unstained. After incubation, the cells were washed twice and resuspended in PBS.

### Transduction of CTLs

The CTL lines were resuspended at 1×10^6^ cells/mL in complete medium, and then incubated with different volumes (0.1, 1, 10, 100 mL) of freshly generated Lenti/hCD40L/E1 for 36 hours at 37°C and 5% CO_2_. To increase the efficiency of transduction, 2 to 3 rounds of transduction were performed. The transduced CTL were subsequently maintained in Yssel medium (Gemini Biological Products, Calabasas, CA) supplemented with 10% serum. Except where stated, cells were transduced at 37°C with 10^3^ vp per cell in Opti-MEM medium. At 6 hours after transduction, the cells were washed in 4 mL PBS and resuspended in fresh medium supplemented with 10% FCS.

### Western blot

CTL were transduced with the Lenti/hCD40L/E1 as described above, and then added into SW1990 cells for 48 h; the total cells were washed with ice-cold PBS and were lysed *in situ* with a buffer containing Tris (40 mM, pH 7.4), 10% glycerol, b-glycerophosphate (50 mM), ethyleneglycol-bis- tetraacetic acid (5 mM), ethylenediaminetetraacetic acid (2 mM), vanadate (0.35 mM), NaF (10 mM), 0.3% Triton X-100, and protease inhibitors (Roche, Penzberg, Germany). After incubation on ice for 30 min, with vortexing every 10 min, cell lysates were centrifuged at 12 000 r.p.m. for 15 min at 4°C. 100 μg of cellular proteins were separated on a 10% SDS-PAGE gel, and the proteins were transferred to the PVDF membranes (Roche). Membranes were blocked with 5% non-fat dry milk in TBST (10 mM Tris-HCl, pH 8.0, 150 mM NaCl, 0.05% Tween 20) and were then incubated with the respective primary antibodies (Adenovirus E1 (1:500) and GAPDH (1:500)) overnight at 4°C. After washing five times for 10 min each in TBST, membranes were incubated with HRP-conjugated secondary antibodies for 2 h, washed again and the peroxidase reaction was performed by an enhanced chemiluminescence detection system to visualize the immunoreactive bands.

### MTT assay

Each group cells were seeded onto a 96-well plate at a density of 5,000–10,000 cells per well for 24 h before serum starvation. After serum starvation for 24 h, cells were incubated in McCOY's 5A medium supplemented with 15% FBS. After 12, 24, 48 or 72 hours, the medium was removed, and MTT reagent was added in each well for a 4-hour incubation at 37°C. Optical densities (OD) at 490 nm were determined through a microplate reader (BIO-TEC Inc, VA). The proliferation rate was calculated as OD (cells plate)/OD (control plate). Values reported here are the averages of triplicate experiments.

### Enzyme-linked immunosorbent assay (ELISA)

2 × 10^5^ CD40L/E1-CTL cells were seeded into 48 well culture plates. And 1 × 10^8^ vp/mL Ad-TRAIL or Ad-GFP virus were added into the culture media. After 48 h, the supernatants were collected and then frozen at −80°C until use. The production of IFN-γ in the supernatants of CTL cells was assessed by ELISA using a commercially available ELISA kit (R&D Systems, Minneapolis, MN, USA) according to the manufacturer's recommendations.

### Effect of CTL on HUVEC tube formation

To evaluate anti-angiogenic effects mediated by Ad-TRAIL-CD40L/E1-CTL, we used the HUVEC tube formation assay. To obtain the new Ad-TRAIL-CD40L/E1-CTL, CTL cells medium was added into Lenti/hCD40L/E1 and Ad5/35-TRAIL with the suitable MOIs 24 hours. Then, HUVEC were cultured in M199 medium in each treatment and their tube formation were evaluated in the microscope.

### Colony formation in soft agar

The soft agar colony formation assays used to assess pancreatic cancer cell proliferation. Each well of a 6-well plate contained 2 mL of 0.5% (w/v) Noble agar (Difco) in DMEM with 10% NBCS. SW1990 cells and the indicated CTL were mixed equally, and 3×10^3^ cells in 2 mL of 0.375% (w/v) Noble agar in 10% NBCS DMEM were added above the polymerized base solution. Plates were incubated (37°C, 5% CO_2_) under standard conditions for 10 days before colony number and diameter were quantified microscopically.

### Animal model and tumor volume measurement

BALB/c athymic nude mice, 4 weeks old, were purchased from Shanghai Experimental Animal Center (Chinese Academy of Sciences, China). SW1990 cell lines were used to grow xenografts in right lower extremity of athymic, nude mice. A group of female athymic nude mice (*n* = 18) was used throughout these studies. On the day of the experiment, mice were anesthetized, and the right lower extremity was exposed. SW1990 Cells were then injected at a constant rate of 2 × 10^6^ cells in a total volume of 100 μl under the hypodermia of right lower extremity. After three days, the mice would be divided to three group, which was the 0.9 NaCl% group (control), CTL group and Ad5/35-TRAIL CTL group. The 100 μl 0.9% NaCl, 100 μl CTL cells (2 × 10^6^ cells) and Ad5/35-TRAIL CTL (2 × 10^6^ cells) were injected into xenografts of right lower extremity in each group mice respectively, and then on alternate days injected again, 4 injections in all. 3 days after tumor cell inoculation, mice were observed and tumor volume was measured (V = a × b^2^/2, a = long diameter of tumor, b = short diameter of tumor). 24 days after tumor cell inoculation, nude mice was sacrificed. Animal care and experiments were carried out in accordance with guidelines of the Xi'an Jiaotong University on Animal Care. In the Ad-TRAIL-CD40L/E1-CTL group, one mice died three days after injection, with no tumor growth.

### CD31 IHC

The xenografts were fixed in 10% neutral buffered formalin and processed in paraffin. Sections were cut on a microtome and mounted on glass slides. Sections were dewaxed and hydrated in graded alcoholic solutions and distilled water.

Immunohistochemical staining for CD31 were performed using the SABC kit according to the manufacturer's instruction. For immunohistochemistry, endogenous peroxidase activity was quenched with 0.5% hydrogen peroxide in methanol for 30 minutes, and non-specific binding was blocked using 5% normal goat serum for 30 minutes. Sections were then incubated with primary antibodies overnight at 4°C and incubated with the appropriate biotinylated secondary antibody for 30 minutes at room temperature. Sections were then incubated with avidin/biotin complex for 30 minutes at room temperature. Following washing in PBS, immunoreactivity was visualized using DAB. Sections were counterstained with hematoxylin, and dehydrated in graded alcohols. For evaluation of CD31 protein expression, the densitometry analysis of immunohistochemical staining was performed using the Image-Pro Plus 4.5 software.

### TUNEL assay

Apoptotic cells were detected using the TUNEL assay. Sections were washed twice with washing buffer, and then 10 mM proteinase K (1:200 dilution) was added for 15 s, followed by incubation with 1 μl terminal deoxynucleotidyl transferase (TdT, 1:10 dilution) and 1 μl DIG-UTP (1:10 dilution) in a humidified box at 37°C for 2 h. Sections were then washed twice in TBS, and covered with remaining liquid at 37°C for 30 min in a humidified box. And then incubated with a biotinylated anti-DIG antibody (1:20 dilution), and incubated for an additional 30 min with SABC (1:100 dilution) at room temperature. Sections were then washed twice in TBS, and 50 μl of a DAB solution modified as described earlier for immunohistochemical staining was applied to each section following TUNEL incubation. Sections were subsequently rinsed in distilled water, counterstained with hematoxylin, washed and dehydrated through graded ethanols, and cleared in xylene. Sections were coverslipped and the results were documented using an Olympus PM-6 microscope equipped with an Olympus digital camera. Quantitative apoptosis analysis of cells was performed by counting the TUNEL-positive cells within a field of 200 cells at 400× magnification.

### Statistical analysis

All statistical analyses were performed using the SPSS18.0 software. The results were presented as the mean ± s.d. of three replicate assays. Differences between the groups were assessed by the analysis of variance (ANOVA) or the nonparametric test, Kruskal Wallis. *P* < 0.05 was considered statistically significant.
